# Notice of Appointment

**Published:** 1884-03

**Authors:** 


Notice of Appointment.—We take the most sincere pleasure in noticing the appointment as Lecturer on Obtetrics in the Spring Course of the Medical Department of the University of Buffalo, of Dr. Joseph W. Keene, of this city. Long and intimate acquaintance has taught us to look upon him as the peer of any of his medical brethren of the city, in all that goes to make a man and a physician.

In his appointment the college has shown its appreciation of his sterling qualities and is to be congratulated in at least equal measure with the newly appointed lecturer.

